# Comprehensive analysis of single-cell RNA sequencing data from healthy human marrow hematopoietic cells

**DOI:** 10.1186/s13104-020-05357-y

**Published:** 2020-11-10

**Authors:** Xin Zhao, Shouguo Gao, Sachiko Kajigaya, Qingguo Liu, Zhijie Wu, Xingmin Feng, Fengkui Zhang, Neal S. Young

**Affiliations:** 1 Hematology Branch, National Heart, Lung and Blood Institute, National Institutes of Health, Bethesda, MD 20892 USA; 2 State Key Laboratory of Experimental Hematology, Institute of Hematology and Blood Diseases Hospital, Chinese Academy of Medical Sciences and Peking Union Medical College, Tianjin, 300020 China

**Keywords:** Single cell RNA-sequencing, Hematopoiesis, Differentiation, Quiescence of stem cell

## Abstract

**Objective:**

Single cell methodology enables detection and quantification of transcriptional changes and unravelling dynamic aspects of the transcriptional heterogeneity not accessible using bulk sequencing approaches. We have applied single-cell RNA-sequencing (scRNA-seq) to fresh human bone marrow CD34^+^ cells and profiled 391 single hematopoietic stem/progenitor cells (HSPCs) from healthy donors to characterize lineage- and stage-specific transcription during hematopoiesis.

**Results:**

Cells clustered into six distinct groups, which could be assigned to known HSPC subpopulations based on lineage specific genes. Reconstruction of differentiation trajectories in single cells revealed four committed lineages derived from HSCs, as well as dynamic expression changes underlying cell fate during early erythroid-megakaryocytic, lymphoid, and granulocyte-monocyte differentiation. A similar non-hierarchical pattern of hematopoiesis could be derived from analysis of published single-cell assay for transposase-accessible chromatin sequencing (scATAC-seq), consistent with a sequential relationship between chromatin dynamics and regulation of gene expression during lineage commitment (first, altered chromatin conformation, then mRNA transcription). Computationally, we have reconstructed molecular trajectories connecting HSCs directly to four hematopoietic lineages. Integration of long noncoding RNA (lncRNA) expression from the same cells demonstrated mRNA transcriptome, lncRNA, and the epigenome were highly homologous in their pattern of gene activation and suppression during hematopoietic cell differentiation.

## Introduction

Hematopoiesis has been modeled as a stepwise process of sequential binary decisions, associated functionally with loss of self-renewal, upregulation of transcription factors, and downstream gene expression characteristics of progenitor cells and their mature progenies [1–4]. Self-renewing HSCs and MPPs are infrequent; oligopotent and unipotent progenitors have briefer life spans, increase numerically, and ultimately, differentiate into mature blood cell types. From MPPs, the common lineages for myelopoiesis (common myeloid progenitor, CMP) and lymphopoiesis (common lymphoid progenitor, CLP) are segregated. In myeloid differentiation, oligopotent CMPs undergo further restriction into bivalent granulocyte-monocyte progenitors (GMPs) of granulocytes and monocytes, and megakaryocyte-erythroid progenitors (MEPs) provide platelets and red blood cells. Hematopoiesis is also highly responsive to environmental alteration, such as blood loss or in confrontation of infectious agents. Under stress, regulation of hematopoiesis involves HSCs to exit from quiescence and entry into differentiation pathways.

Methods utilized for decades to define events in blood production depend on considerable manipulation of cells in the laboratory [physical stresses of temperature changes, altered gravity during centrifugation, and ambient oxygen concentrations much higher than in bone marrow (BM)]. These methods are convenient, but their experimental conditions often predicate on existing models. Novel methods, which are able to interrogate single cells, to generate vast amounts of data from each cell, and to minimally manipulate specimens have challenged established models of hematopoiesis. Progenitor populations have been revealed as highly heterogeneous in both developmental stages and fate potentials [5–13]. The marrow hematopoietic hierarchy of adults appears dominated by two progenitor classes (multipotent and unipotent progenitors) over scarce oligopotent progenitors [8].

Previously we used single cell RNA sequencing (scRNA-seq) of fresh BM from healthy donors as a reference to identify aneuploidy cells [14]. In the current analysis, we had a comprehensive analysis to examine the presence of dynamic aspects of the transcriptional heterogeneity. Our data revealed a continuum of transition states among the progenitor groups. Further integrative analysis with lncRNA and scATAC-seq data proved the collaboration of transcriptome and epigenome during hematopoietic differentiation.

## Main text

### Methods

#### Subjects, samples, scRNA-seq, and quantitative RT-PCR

Sample collection and scRNA-sequencing were described in previous study [14]. BM was collected from healthy donors, and Lin(CD3CD14CD19)^−^CD34^+^CD38^−^ and Lin(CD3CD14CD19)^−^ CD34^+^CD38^+^ populations were sorted. SMARTer RNA amplification was performed with C1 Fluidigm, and libraries were sequenced with HiSeq-2500. Lineage-specific mRNAs and two housekeeping genes were pre-amplified and analyzed using quantitative RT-PCR.

#### Data analysis

**Fig. 1:**
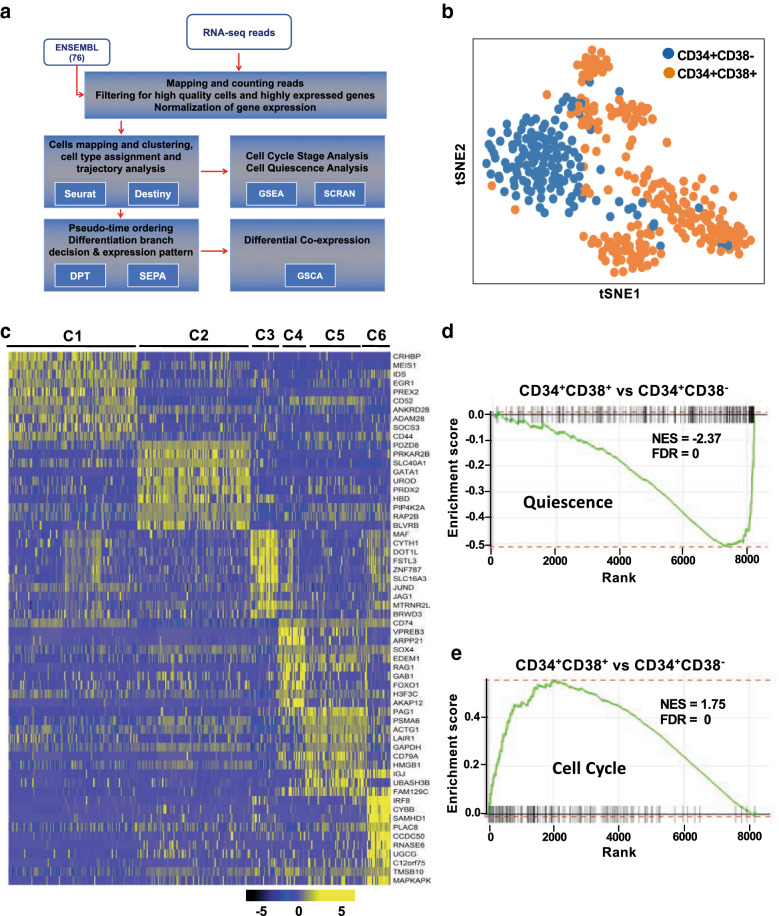
Heterogeneity of hematopoietic stem and progenitor cells quantified by scRNA-seq. **a** Schematic of bioinformatics analysis workflow to analyze scRNA-seq data. **b** A t-distributed Stochastic Neighbor Embedding (tSNE) plot of single-cell gene expression data. Each dot represents one cell. Cells were labelled based on expression of a surface marker CD38. **c** Unsupervised hierarchical clustering of gene expression data for all cells. Clustering was performed by using all 2093 variable genes across all cells. Top 10 genes (row) enriched in each cluster (column) are displayed in a heatmap, showing gene expression on a log2 scale from black to yellow (low to high). **d** A GSEA plot shows decreased expression of a gene set involved in cell quiescence in Lin^−^CD34^+^CD38^+^ cells vs Lin^−^CD34^+^CD38^−^ cells. **e** A GSEA plot represents increased expression of a gene set involved in cell cycling in Lin^−^CD34^+^CD38^+^ cells relative to Lin^−^CD34^+^CD38^−^ cells

The detailed workflow is presented in Fig. 1a. In brief, Subread was used to align reads to the human hg19 genome and featureCounts to assign reads to genes using ENSEMBL annotation. Quiescence of CD38^−^ cells were characterized with GSEA. Highly variable genes (HVGs) across single cells that identified by the Seurat were applied to PCA and tSNE, and cells were clustered with DBSCAN. HSPC type was assigned to each cluster based on the overlapping significance between HSPC- and cluster-specific genes. Dimensionality reduction was also performed using diffusion map [15], implemented in the destiny R package.

Gene set co-expression analysis (GSCA) [16] allowed calculation of pairwise correlations within a gene set, in three branches, which formed three distinct correlation vectors. The Euclidean distance of the three correlation vectors was calculated to determine differential co-expression of a predefined gene set.

We downloaded raw data for GSE75478 [11] from the GEO repository, in which RNA sequencing was applied to ~ 1000 sorted HSPCs. Expression of lncRNAs annotated in Gencode was calculated. We checked expression lineage specificity of lncRNA neighboring mRNAs (< 50,000 bases) to identify their co-operation in differentiation. The single-cell assay for transposase-accessible chromatin by sequencing (scATAC-seq) profiles of ~ 2000 cells with different hematopoietic cell types [17] were downloaded.

### Results

We enriched cells in the more primitive Lin(CD3CD14CD19)^−^CD34^+^CD38^−^ compartment and more differentiated Lin(CD3CD14CD19)^−^CD34^+^CD38^+^ compartment for scRNA-seq with an average depth of 5–20 million read pairs [18]. Overall, 391 cells across four donors were retained for further analyses.

#### Cellular diversity and differentiation trajectories in HSPCs

As our results of this part were comparable to previous studies, including cell populations and differentiation trajectories [7, 14, 15, 17], the results were only briefed here and the details were shown in supplemental results (Additional file 1). Note that our analysis was more comprehensive and adopted more approaches (Seurat, SEPA, destiny, PAGA, and in-house programs) than previous studies, thus provided a complete view of hematopoiesis from different angles. Firstly, our analysis provided important confirmation and extension of scRNA-seq work for understanding hematopoietic hierarchy. Secondly, instead of examining the expression of a few known hematopoietic genes, our study characterized expression patterns of around 30 membrane marker genes, transcription factors, and lineage-specific mRNAs. Statistical analysis with SEPA further extended the gene list involved in hematopoiesis. Thirdly, in this study, expressions of some genes in the same cells were validated with RT-PCR. Our analysis provided a relatively complete view to hematologists.

Single cells within Lin^−^CD34^+^CD38^−^ and Lin^−^CD34^+^CD38^+^ compartments showed fundamentally different gene expression profiles (Fig. 1b, Additional file 2: Figure S1A). Clustering analysis allowed us to create a detailed map that included six transcriptionally homogeneous subpopulations (Fig. 1c). Trajectory analysis revealed an early split of fate decisions from HSCs towards erythroid/megakaryocytes and myelo/lymphoid cells, which separated further into lymphoid and neutro/monocyte progenitors (Additional file 3: Table S1). Several well-characterized transcriptional factors essential for lineage commitment displayed strong dynamics in one or more of the lineages, distinct among lineage trajectories (Additional file 2: Figure S5A). A list of top genes that were dynamically expressed along with differentiation from stem cells to different progenitors was shown in Additional file 4: Table S2.

#### Quiescence of hematopoietic stem cells

A key component and important driver of transcriptional heterogeneity and cell decision processes is the cell cycle [19, 20]. Quiescence is a fundamental characteristic of hematopoietic stem cells, as most of them reside in G0; quiescence is believed to protect HSCs from functional exhaustion and biochemical insults [21]. We anticipated that genes related to quiescence should be active in the stem cell population and genes related to cell cycle to be inactive. We first sought to dissect cell cycle states between the two major populations of Lin^−^CD34^+^CD38^−^ and Lin^−^CD34^+^CD38^+^ cells. When the complete differential gene expression dataset was submitted to Gene Set Enrichment Analysis, Lin^−^CD34^+^CD38^+^ cells displayed decreased expression of quiescence-related genes (Fig. 1d, FDR = 0) and enhancement of cell cycle genes (Fig. 1e, FDR = 0), compared to Lin^−^CD34^+^CD38^−^ cells. We next took advantage of a recently reported predictive algorithm for allocating individual cells to G0/G1, S, and G2/M cell cycle categories based on single-cell transcriptomes [22]. Distribution of single cells across these three cell cycle categories was in agreement with enrichment of cell cycle terms in genes upregulated in CD38^+^ subpopulations (Additional file 2: Figure S1C). Such a large-scale transition of cells to S and G2/M phases with differentiation was consistent with other reports [23–25], and supported the validity of our single-cell results.

#### Identification of differentially co-expressed pathways with differentiation

Genes do not function independently but rather interact in concert through a complex regulatory network. We sought to identify differences in co-expression patterns at different hematopoietic cell stages. We employed GSCA, which systematically identifies differentially co-expressed modules between stem cells and lineage differentiation populations. Application of GSCA on the KEGG pathway sets revealed that certain pathways were differentially co-expressed at different hematopoiesis stages (Additional file 5: Table S3).

**Fig. 2:**
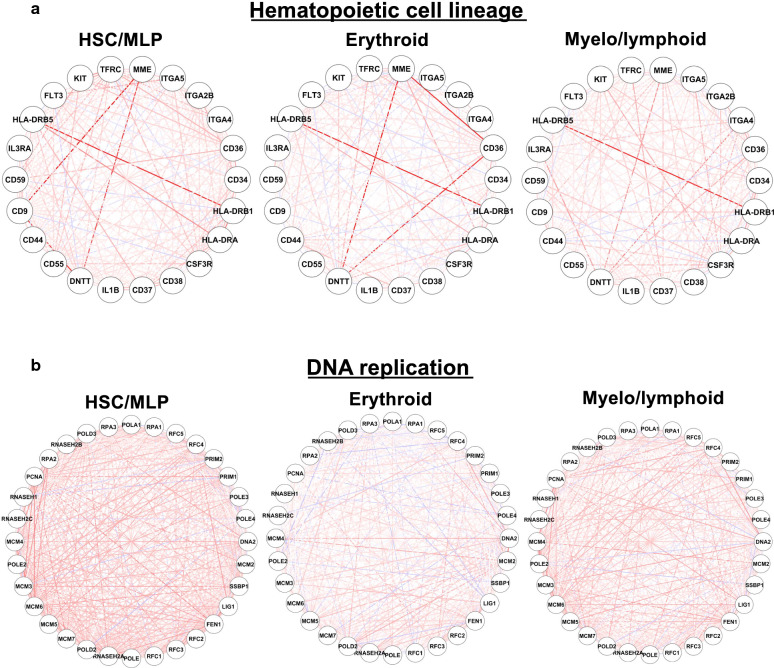
Transcriptional regulatory network models for differentiation from HSCs to MEPs or myelo/lymphoids. Transcriptional networks demonstrate biological relevance of genes involved in the hematopoietic cell lineage pathway (**a**) and the DNA replication pathway (**b**). Correlation and anti-correlation are indicated with red and blue lines, respectively

The top differentially expressed pathways mostly related to hematopoiesis. The most differentially co-regulated pathway among the three branches was the hematopoietic cell lineage (*p* < 0.001, DI = 0.146); in detail, co-expression relationships in the network displayed specific regulatory alteration or conservation (Fig. 2a). For example, co-expression between *HLA-DRB*5 and *HLA-DRB1* was conserved during differentiation. Co-expression between *MME* and *DNTT* was strong in MEP, consistent with downregulation of both genes during erythroid differentiation; decreased co-expression of *MME* and *DNTT* in myelo/lymphoid could be attributed to diverse expression levels in myeloid cells and lymphoid cells. Co-expression between *CD36* and other genes including *DNTT* and *MME* was apparent along the erythroid developmental pathway. DNA-replication related genes exhibited decreasing co-expressed patterns in both erythroid and myeloid populations (Fig. 2b), indicating decreased DNA replication activity with cell differentiation and these genes were correlated with this process.

#### lncRNAs are correlated with differentiation

**Fig. 3:**
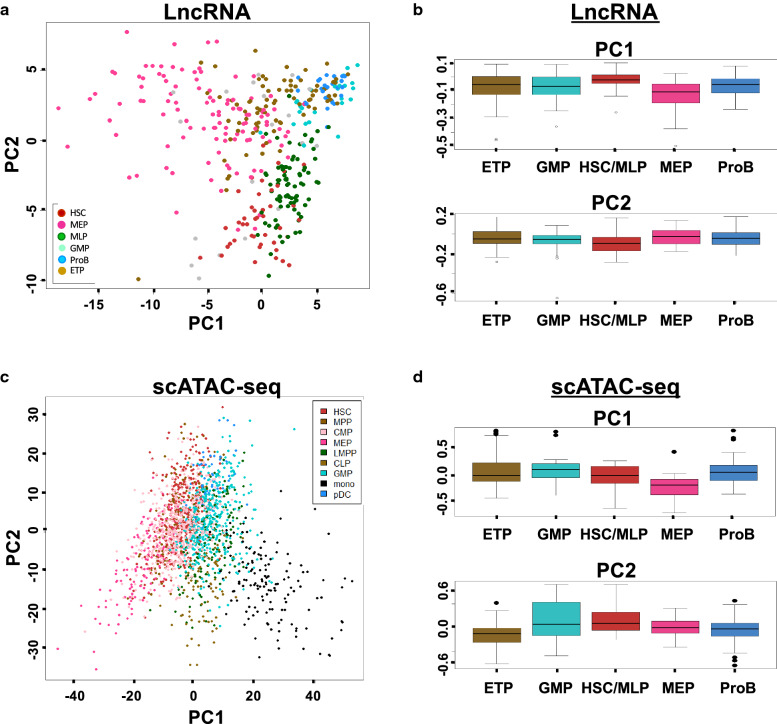
Early fate transitions in human BM CD34^+^ progenitors. **a** PCA plot of lncRNA expression from our scRNA-seq data. Highly variable lncRNAs were used for analysis. Each dot indicates one cell. **b** Projection of transcriptomic lncRNA gene modules onto scRNA-seq data in **a**. LncRNAs that neighbor the cluster-specific genes (generated from Fig. 1b) on a chromosome were used for analysis. Each dot represents a neighboring lncRNA. Vertical lines (low to high): first, median, and third quartiles. **c** PCA plot of scATAC-seq data from Buenrostro et al. [17]. Each dot indicates one cell. **d** Projections of five transcriptomic gene modules onto scATAC-seq PCA in **c**. ATAC-seq transcriptional factor scores of the cluster-specific genes on chromosome were used for analysis. Each dot represents a transcriptional factor. Modules were segregated into two groups, with either significantly positive scores on PC1 or PC2 that were consistent with transcriptional dynamics in Fig. 2

lncRNAs are defined as a subclass of noncoding RNAs that are longer than 200 nucleotides lacking protein coding capacity [26]. They have emerged as novel regulators of gene expression, transcriptionally and post-transcriptionally. lncRNAs are expressed in a cell type-specific manner and control the development of several lineages in the hematopoietic system and immune response [26]. We applied principal component analysis (PCA) using highly variable lncRNAs in our dataset (Fig. 3a) and expression patterns of lncRNAs in single CD34^+^ cells were highly stage- and lineage-specific. Principal component 1 showed two different branches from HSCs towards erythroid/megakaryocyte and myeloid/lymphoid, respectively; principal component 2 reflected the difference between HSCs and their progenies.

We further projected lncRNAs to their neighboring genes to check consistency of results from mRNA and those from lncRNA (Fig. 3b). Principal component is the weighted linear combination of the initial variables, thus the contribution of each lncRNA on principal component can be represented by the weight. We used lncRNAs located adjacent to cluster-specific genes (neighbors on a chromosome) for analysis. These cluster-specific genes neighboring lncRNAs were clearly characterized by their contributions to the first two principal components. For example, MEP specific genes’ neighboring lncRNAs contribute to negative side of principal component 1 and HSC/MLP specific genes’ neighboring lncRNAs contribute to negative side of principal component 2. Analysis of published data [11] (Additional file 2: Figures S7A and S7B) yielded similar results, demonstrating the data quality and the robustness of our integrative approach. Thus lncRNAs and their neighboring mRNAs are co-expressed directionally along lineage differentiation pathways.

#### Epigenetic changes during molecular transitions

Epigenetic changes, particularly chromatin remodeling, are primary determinants of cellular potential. Integrative analysis of single-cell transcriptomics and chromatin accessibility should provide insights into regulatory features and the dynamics of human hematopoiesis [17]. We examined a recently published scATAC-seq dataset, which identifies regions of open or active chromatin regions of human BM hematopoiesis [17], in order to integrate chromatin dynamics and our transcriptional model.

To investigate whether the hematopoietic hierarchy calculated from our transcriptomic data was reflected in chromatin accessibility, we downloaded the transcriptional factor motifs accessibility score, as inputs for PCA across progenitor types. PC analysis and pseudotemporal ordering of the scATAC-seq data showed distinct differentiating patterns of hematopoiesis (Fig. 3c; Additional file 2: Figure S7C). PC1 and PC2 showed the structure of our predicted hematopoietic hierarchy, indicating mainly two branches of differentiation from the stem cell, one towards erythroid cells/megakaryocytes and the other to myeloid/lymphoid cells (Fig. 3c). With the same approach as lncRNAs, when we projected scATAC-seq transcriptional factors to genes from our transcriptomic clusters on to this PCA, MEP-dependent genes and myeloid/lymphoid-dependent genes were located on opposing sides of the PC1 axis with same direction (Fig. 3d). scATAC-seq transcriptional factors for “primed” genes exhibited high levels of accessibility in early progenitors, with HSCs and MLPs exhibiting multilineage epigenetic priming for these loci as well. In a previous study, projection of hematopoietic lineage specific genes on PCA plot with bulk ATAC-seq data showed MEP and myeloid/lymphoid genes were located on opposing sides of one principal component [12]. Though using the same analytical procedures, our analysis is more comprehensive because we used scATAC-seq data and included the same cell lncRNA data. Therefore, intermediate stages exhibited evidence for multilineage priming at both transcriptomic and epigenetic levels. Thus, we confirmed that scATAC-seq data allowed identification of cell types, and further that signature genes and their associated promoters’ chromatin accessibility, as well as neighboring lncRNAs [26], aligned well with cell differentiation (Fig. 3).

## Limitations

One analysis is limited with small number of cells and some drop-seq platforms can profile tens of thousands of cells. However Smart-seq2 C1 platform is valuable for detection of more genes per cell (~ 9000 genes) than 10× platform (~ 2000 genes, high dropout rate) [27]. Our dataset is suitable for co-expression analysis because dropout events dramatically affect correlation calculation [28].

We integrated scRNA-seq and scATAC-seq datasets from different studies. Profiling expression and chromatin accessibility in same cells is more powerful to examine the relationship between genome structure and patterns of gene expression [29].

## Supplementary information

**Additional file 1.** Supplemental methods and results.

**Additional file 2: Figure S1.** (**A**) t-distributed Stochastic Neighbor Embedding (tSNE) plot of single-cell gene expression data. Cells were labeled according to assigned cell types. (**B**) Assignment of a HSPC type to each cluster based on the significance of overlapping between HSPC- and cluster-specific genes (Fisher’s exact test). (**C**) Proportions of HSCs, MLPs, GMPs, ProBs, ETPs, and MEPs in each of the cell cycle categories. Cell types displayed were based on the tSNE results. **Figure S2.** (**A**, **B**) Visualization of the HSPC continuum. Cells were colored based on FACS sorting surface marker CD38 in (**A**). Clusters 2 and 6, and undefined cells were hidden to show Cluster 3 clearly in (**B**). (**C**) Expression levels of immunophenotypic populations based on surface markers were overlaid on the cellular hierarchy. (**D**) Enriched GO terms of differentially expressed genes in ETPs. **Figure S3.** Visualization of the HSPC continuum. Ordering of individual cells into a three-dimensional independent component space of hematopoietic lineages using a diffusion map. Each ball represents one cell. Cells were colored based on different clusters defined from Fig. 1c in Panel **A**. (**B**) PCA of single-cell gene expression data. Cells were labeled according to assigned cell types. (**C**) Partition-based graph abstraction generated a topology-preserving map of single cells. Nodes correspond to cell groups and edge weights quantifies the connectivity between groups. **Figure S4.** Large-scale shifts in gene expression during development of hematopoietic cells. (**A**) Global analysis of gene expression kinetics along the trajectory identified genes that varied significantly over pseudotime development. Bars on top indicate locations of individual cells, colored by stages of development, along this developmental trajectory. (**B**) Enriched GO terms of differentially expressed genes in each population. **Figure S5.** Reconstructing the topology of early fate decisions. (**A**) Expression levels of hematopoietic transcriptional factors were overlaid on the cellular hierarchy. (**B**) Kinetic diagrams show expression of known markers of different developmental stages over the developmental progression. Dots indicate individual cells colored according to developmental stages. **Figure S6.** Quantitative RT-PCR analysis of expression of signature mRNAs. (**A**) Expression of lineage specific genes measured using single-cell qPCR. (**B**) Correlation of the expression of lineage specific genes measured by different methods. X and Y axes represent expression levels measured using scRNA-seq and single-cell qPCR, respectively. Each dot indicates a cell. **Figure S7.** The raw data for GSE75478 [11] were downloaded from the GEO repository, in which ~ 1000 sorted HSPCs were subjected to RNA sequencing. Using the data, lncRNAs annotated in Gencode was calculated with subreads and featureCounts. PCA analysis was subjected to assess whether lncRNA could identify hematopoietic populations and contribution of each lncRNA. Subsequently, lncRNA neighboring mRNAs (< 50,000 bases) were examined to elucidate their co-operation in differentiation. (**A**) PCA of lncRNA from Velten’s scRNA-seq data. Each dot indicates one cell. (**B**) Projection of transcriptomic lncRNA gene modules onto scRNA-seq data in (**A**). Each dot represents a lncRNA. Vertical lines (low to high): first, median, and third quartiles. (**C**) Ordering of individual cells from Buenrostro et al. [17] using a diffusion map. scATAC-seq profiles of ~ 2000 cells with different hematopoietic cell types (HSC, MPP, CMP, MEP, LMPP, CLP, GMP, mono, and pDC) were downloaded. The downloaded transcription factor motif accessibility scores were subjected to PCA and diffusion map to investigate whether chromatin accessibility landscape could characterize differentiation trajectories of human hematopoiesis. Further, cell type expression specificity of transcriptional factors was examined to identify consistency between epigenetic and transcriptomic data, by assuming that lineage specific transcriptional factors are activated through having their promoter regions accessible in certain differentiation lineages.

**Additional file 3: Table S1.** GO terms of genes dynamically changed along hematopoietic lineage differentiation.

**Additional file 4: Table S2.** Top 50 genes dynamically expressed along pseudotime ordering.

**Additional file 5: Table S3.** KEGG overlap pathways in co-expression analysis.

## Data Availability

The datasets generated and analysed during the current study are available in the GEO repository with accession number GSE99095 (https://www.ncbi.nlm.nih.gov/geo/query/acc.cgi?acc=GSE99095).

## Abbreviations

BM: Bone marrow
scRNA-seq: Single-cell RNA sequencing
scATAC-seq: Single-cell assay for transposase-accessible chromatin sequencing
lncRNA: Long noncoding RNA
HSPC: Hematopoietic stem and progenitor cell
MPP: Multipotent progenitor
PCA: Principal Component Analysis
CMP: Common myeloid progenitor
CLP: Common lymphoid progenitor
GMP: Granulocyte-monocyte progenitor
MEP: Megakaryocyte-erythroid progenitor
GSCA: Gene set co-expression analysis
