# Supplementary material for: Comprehensive analysis of single-cell RNA sequencing data from healthy human marrow hematopoietic cells
Source: BMC Res Notes. 2020 Nov 10;13:514. doi: 10.1186/s13104-020-05357-y (PMC7653854; doi:10.1186/s13104-020-05357-y)
Supplement: Supplementary file 1 — Additional file 1. Supplemental methods and results. [file 13104_2020_5357_MOESM1_ESM.docx]

**ADDITIONAL METHODS**

**Subjects and samples**

BM was collected from four self-declared healthy donors (31/M, 34/M, 57/F, and 58/M) in accordance with the Declaration of Helsinki and after enrollment in research protocols approved by the Institutional Review Board of the National Heart, Lung, and Blood Institute. BM was collected into syringes containing media supplemented with heparin. Lin(CD3CD14CD19)^-^CD34^+^CD38^-^ and Lin(CD3CD14CD19)^-^ CD34^+^CD38^+^ populations were sorted using the LSRII Fortessa Cytometer (BD Biosciences), as previously described [1].

**scRNA-seq**

Freshly prepared cells without cryopreservation were subjected to scRNA-seq. Full-length cDNA libraries were prepared as previously described [1]. In brief, the C1 Single-cell Auto Prep System (Fluidigm) was used to perform SMARTer (Clontech) whole transcriptome amplification (WTA) on as many as 96 individual cells. WTA products weres then converted to Illumina sequencing libraries using Nextera XT (Illumina). Each sample was sequenced on the Illumina HiSeq 2500 platform using a 75-bp paired-end sequencing strategy.

**Quantitative RT-PCR**

cDNA from WTA products from 391 single CD34^+^ cells was subjected to gene expression analysis. Lineage-specific mRNAs and two housekeeping genes (*GATA1*, *CD79A*, *CXCR4*, *MEIS1*, *CRHBP*, *GAS5*, *HBD*, *MLLT3*, *MPO*, *NEAT1*, *SNORA76*, *SPINK2*, *TFRC*, *URDO*, *GAPDH*, and *ACTB*) were pre-amplified and analyzed using quantitative RT-PCR following the manufacturer’s protocol (Fluidigm). Gene expression preamplification was performed with Fluidigm Preamp Master Mix (Fluidigm) and TaqMan Assays (Thermofisher). Subsequently, gene expression analysis was performed in a 96.96 quantitative PCR Dynamic Array on the Fluidigm Biomark instrument using Fast TaqMan Assays (Thermofisher), as described previously.^15^ Single-cell gene expression data were first analyzed with the Fluidigm Data Collection software. Expression values over the cutoff of the machine (CT value > 27) were set to 28. After filtering, ΔCT values were calculated by cell-wise normalization to a mean expression level of two housekeeping genes (*ACTB* and *GAPDH*) through subtracting of their mean CT values.

**Data analysis**

The detailed workflow is presented in Fig. 1A. In brief, Subread version 1.4.4 was used to align reads to the human hg19 genome and featureCounts to assign reads to genes using ENSEMBL annotation (version 76). We filtered single-cell transcriptomes with ≥ 2 million reads and removed batch effect using SVA software (SVA Software).

Highly variable genes (HVGs) across single cells that identified by the Seurat package with a z-score cutoff of 0.5 were applied to Principal Component Analysis (PCA). Data dimensions were further reduced with tSNE and cells were clustered with DBSCAN. Dimensionality reduction was also performed using diffusion map [2], implemented in the destiny R package with centered cosine distance. Cells were clustered using t-distributed Stochastic Neighbor Embedding (tSNE) and DBSCAN on highly variant genes, and an HSPC type was assigned to each cluster based on the significance in overlapping between HSPC- and cluster-specific genes. The distributed prediction tracking (DPT) [3], an efficient algorithm to measure transitions between cells using diffusion-like random walks was utilized to estimate the pseudotimes of all cells (https://bioconductor.org/packages/release/bioc/html/destiny.html).

Gene set co-expression analysis (GSCA) [4] allowed calculation of pairwise correlations within a gene set, in three branches, which formed three distinct correlation vectors. The Euclidean distance of the three correlation vectors was calculated to determine differential co-expression of a predefined gene set (the KEGG pathway in our study). We used the “average stage expression” method [5] to assign cell cycle stage. Specifically, we defined four cell cycle scores (G0/G1, S, G2/M, and G2) as average expression (log2(TPM+1)) of phase-specific subsets of the cell cycle genes, as defined in transcriptome of quiescent CD34 [6] and synchronized HeLa cells [7]. A cycle stage on each cell was assigned to that with the highest score.

We downloaded raw data for GSE75478 [8] from the GEO repository, in which RNA sequencing was applied to ~1000 sorted HSPCs. Expression of long noncoding RNAs (lncRNAs) annotated in Gencode was calculated with subreads and featureCounts. PCA analysis was utilized to examine whether lncRNA could identify hematopoietic populations and calculate contributions of each lncRNA on the first three components. Then we checked expression lineage specificity of lncRNA neighboring mRNAs (< 50000 bases) to identify their co-operation in differentiation [9]. The lncRNAs quantification of cells in this study was obtained with the same approach.

The single-cell assay for transposase-accessible chromatin by sequencing (scATAC-seq) profiles of ~2000 cells with different hematopoietic cell types (HSC, MPP, CMP, MEP, LMPP, CLP, GMP, mono, and pre-dendritic cell [pDC]) [10] were downloaded and PCA and diffusion map were applied to downloaded transcription factor motif accessibility scores in order to examine the chromatin accessibility landscape related to differentiation trajectories of human hematopoiesis. Finally, we examined cell type expression specificity of transcriptional factors to identify consistency between epigenetic and transcriptomic data, as it is assumed that lineage specific transcriptional factors are activated promoter accessibility.

**ADDITIONAL RESULTS**

To systematically characterize global transcriptional landscapes of individual cells and early events in hematopoiesis, we enriched cells in the more primitive Lin(CD3CD14CD19)^-^CD34^+^CD38^-^ compartment and more differentiated Lin(CD3CD14CD19)^-^CD34^+^CD38^+^ compartment for scRNA-seq. We sequenced each sample to an average depth of 5 - 20 million read pairs, achieving stable single-cell expression estimation [11]. Overall, 391 single cells across four donors were retained for further analyses. In total, 11995 protein coding genes were detected across all cells, and 4560 genes were assigned to each cell on average. Log (TPM+1) was used to represent gene expression.

**Unsupervised identification of cellular diversity in human BM HSPCs**

Global gene expression analysis of single cells within Lin^-^CD34^+^CD38^-^ and Lin^-^CD34^+^CD38^+^ compartments showed fundamentally different transcriptomes (Fig. 1B, Fig. S1A). Clustering analysis allowed us to create a detailed map that included six transcriptionally homogeneous subpopulations (Fig. 1C). To determine if the clusters we identified contained known progenitor subpopulations, we first identified cluster-specific genes, followed by comparison of this signature to a microarray reference dataset of bulk expression profiles of separated populations [12] (Fig. S1B). We assigned Cluster 1 to an HSC/MLP identity, based on highly expressed genes typical for both subpopulations [13]. Cluster 2, in which erythroid fate regulators and markers GATA1, CD36, and KLF were highly expressed, was assigned to MEP. Cluster 3 expressed genes that are highly expressed in both defined GMP and Early B, probably representing an early stage of myeloid/lymphoid progenitors. Clusters 4, 5, and 6 were assigned to B lymphoid progenitors (ProBs) and early T cell progenitors (ETPs), respectively. Cluster 6 that also highly expressed the myeloid marker IRF8 probably represented neutro/monocyte progenitors.

A key component and important driver of transcriptional heterogeneity and cell decision processes is the cell cycle [14, 15]. Quiescence is a fundamental characteristic of hematopoietic stem cells, as most of them reside in G0; quiescence is believed to protect HSCs from functional exhaustion and biochemical insults [16]. We anticipated that genes related to quiescence should be active in the stem cell population and genes related to cell cycle to be inactive. We first sought to dissect cell cycle states between the two major populations of Lin^-^CD34^+^CD38^-^ and Lin^-^CD34^+^CD38^+^ cells. When the complete differential gene expression dataset was submitted to Gene Set Enrichment Analysis, Lin^-^CD34^+^CD38^+^ cells displayed decreased expression of quiescence-related genes (Fig. 1D, FDR = 0) and enhancement of cell cycle genes (Fig. 1E, FDR = 0), compared to Lin^-^CD34^+^CD38^-^ cells. We next took advantage of a recently reported predictive algorithm for allocating individual cells to G0/G1, S, and G2/M cell cycle categories based on single-cell transcriptomes [5]. Distribution of single cells across these three cell cycle categories was in agreement with enrichment of cell cycle terms in genes upregulated in CD38^+^ subpopulations (Fig. S1C). Such a large-scale transition of cells to S and G2/M phases with differentiation was consistent with other reports [17-19], and supported the validity of our single-cell results.

**Developmental trajectories in early human hematopoiesis**

For a detailed analysis of the transition from stem cells to lineage-restricted progenitors, we used Destiny to arrange each cell by pseudotemporal ordering based on gene expression [2]. Lineages clearly separated among Lin^-^CD34^+^CD38^+^ progenitors (Fig. S2A). For purposes of visualization, we tinted trajectories based on identified clusters, and four separate branches originated from Cluster 1 (HSC/MLP; Fig. S3A). A proportion of cells within the stem cell compartment were primed toward MLPs, and the earliest fate split separated erythroid-megakaryocyte progenitors from lymphoid-myeloid progenitors which separated further into lymphoid and neutro/monocyte progenitors (Fig. S3A and S3B). Cells in Cluster 3 (GMP/Early B) represented an intermediate state of lymphoid/myeloid differentiation (Fig. S2B, Fig. S3B).

Clustering assumes that data are composed of biologically distinct groups such as discrete cell types or states, while pseudotemporal orderings assumes that data lie on a connected manifold. To unify both viewpoints and estimate the relationship of single HSPCs, we further used Partition-based graph abstraction (PAGA), which provides a graph-like map of cells that preserves both continuous and disconnected structures in data at multiple resolutions [20]. This method allowed us to trace a putative biological process as from a progenitor to terminal fates in a way that is robust to spurious edges. PAGA captured some of the main features of hematopoiesis, such as proximity of HSCs and MLPs, as well as a close connection between HSCs and MEPs (Fig. S3C), consistent with our Destiny and PCA results. Downstream of MLP was GMP, an intermediate state that further differentiated towards ProB and ETP.

To determine how well a stable state reflected classification of cells sorted based on surface membrane markers, we estimated the expression of CD34 and CD38 as well as other surface antigens commonly utilized for cell type identification (CD45RA, CD49f, CD10, CD36, CD41, and CD135) [12] along our imputed trajectories (Fig. S2C; expression of CD7, CD90, and CD1a were unreliably low in our dataset). Despite presumed temporal discordance between mRNA and protein production (surface protein levels lag changes in mRNA levels), lineage commitment markers were well distributed along differentiating trajectories. As expected, CD34 decreased in all lineages, consistent with a previous report of CD34 downregulation as cells commit to differentiation [21]. The erythroid marker CD36 and the megakaryocyte marker CD41 were exclusively expressed along Branch 2. CD135 or FLT3, expression of which on HSPCs has been interpreted to indicate lympho-myeloid (granulocyte, monocyte, B cell, and T cell) potential and loss of the ability to differentiate towards erythroid and megakaryocyte lineages [22], was highly expressed in Branches 4 and 5. Cells in Branches 4 and 5 were both assigned to ETPs, the transcriptome of which closely resembled that of myeloid progenitors [12]. The *MME* gene (encoding CD10 in human) was highly expressed in Branches 3 and 4, indicating lymphoid fate of these progenitors [23]. Thus, we postulated that Branches 3 and 4 differentiated towards lymphocytes and Branch 5 towards myeloid cells. The location of a specific HSPC population in the diffusion map was consistent with known lineage relationships between mature cell types and their respective progenitors. In contrast to these genes, we did not find expression of any specific surface protein identified a very early stage of HSPCs, reflecting limitation of cell surface makers in defining human stem and early progenitor cells.

Taken together, we reconstructed the lineage relations of hematopoiesis, revealing an early split of fate decisions from HSCs towards erythroid/megakaryocytes and myelo/lymphoid cells, which separate further into lymphoid and neutro/monocyte progenitors.

**Large-scale shifts in gene expression during hematopoietic development**

Differentiation generally involves specifically regulated gene expression. To understand the dynamics of transcriptional changes during hematopoietic differentiation, we examined trends in gene expression in each of the four branches. Clusters of genes altered in expression during specific developmental stages, suggesting sequential large and coordinated changes in gene expression hematopoiesis (Fig. S4A). From gene ontology, erythroid cells showed enrichment of upregulated genes involved in hemoglobin synthesis (Fig. S4B); lymphoid/myeloid progenitors showed increased expression of immune responses and B-cell development pathway genes, and higher expression of cell adhesion, responses to virus and bacteria (Fig. S4B, Fig. S2D, and Table S1). Our analysis also revealed a major contribution of cell cycle-associated genes to the upregulated erythroid genes. These four mapped differentiation trajectories were therefore consistent with our current understanding of early hematopoiesis, indicating that pseudotime reconstruction provides a powerful method to chart the dynamic processes that underlie early HSPC differentiation at single cell resolution.

Several well-characterized transcriptional factors essential for lineage commitment displayed strong dynamics in one or more of the lineages, with clear differences among lineage trajectories (Fig. S5A). Due to essential roles in hematopoiesis of these genes, we applied qPCR to validate the scRNA results. There were significant correlations (with r between -0.36 and -0.9) between the ddCt of single cell qPCR and the log2(TPM ) of scRNA-Seq data (Fig. S6A). Expression changes of these transcriptional factors measured by qRT-PCR along the differentiation trajectory were illustrated in S6B Fig. For example, expression of *MEIS1* and *HLF* peaked in Lin^-^CD34^+^CD38^-^ cells and decreased along other trajectories. Erythrocyte as well as megakaryocyte development is strictly dependent on the transcription factors, such as GATA2, GATA1, KLF1, and TAL1 [24-26], which were dynamically expressed from Branch 1 towards Branch 2. KLF1 is a known regulator of early erythroid precursor genes, as well as a suppressor of the megakaryocyte lineage. Loss of GATA1 leads to complete loss of erythropoiesis*. GATA2* and *TAL1* expression have not only been reported in erythroid and megakaryocyte-committed progenitors but also in multipotent lympho-myeloid populations with erythroid and megakaryocyte potential [27, 28]. Thus, any multipotent HSPC downstream of HSCs with erythroid and megakaryocyte potential would be expected to express these genes, as shown in our data. Recently, several groups have suggested, based on functional studies that megakaryocytes originate directly from multipotent cells rather than from oligopotent progenitors like CMP [8, 29]. However, we did not observe a distinct branch that could be assigned to megakaryocyte progenitors, likely attributable to low cell numbers and infrequency of megakaryocyte progenitors in the HSPC population. Of interest, one well-known megakaryocyte marker, CD41 (ITGA2B), was highly expressed in Branch 2, consistent with bipotential cells capable of erythroid and megakaryocyte differentiation. The myeloid cell maturation marker IRF8 was exclusively expressed in Branch 5, along with the neutrophil marker CSF3R, consistent with the fate of cells in Branch 5 to monocyte and neutrophil differentiation. *EBF1* was mainly activated along the other two trajectories, Branches 3 and 4, indicating lymphocyte potential.

In kinetic diagrams, upregulation of well-known genes related to the specific function of the relevant cell types in pseudotime ordering was in accordance with their early and late development stages (Fig. S5A). The majority of cells characterized as erythroid dynamically expressed genes such as *KLF1*, *GATA1*, *TAL*, and *NFIA*. Expression of *GATA2* was high in progenitor and decreased gradually with erythroid maturation, while *GATA1* expression was not detected in early cells but only expressed in late erythroid cells. GATA1 and GATA2 belong to the same TF family and share the same binding motif. Thus, switching of GATA factors is a well characterized mechanism during erythroid differentiation and our data suggest that GATA2 drives erythroid lineage specification with GATA1 taking over from GATA2 during lineage commitment. Similarly, cells differentiating to lymphocytes dynamically expressed *DNTT*, *EBF1,* and *HHIP*. Genes like *LGMN*, *IRF8*, *IRF7*, and *LGALS1* were highly expressed in neutrophil/monocyte progenitors. *PU.1* (*SPI1*) is a known myeloid specifier but was only low expressed in a few cells in our dataset. *MPO*, *ENO1*, and *SPINK2* expression was gradually increased in Branch 4. Thus, cells in Branch 4 showed both the signature of immature myeloid and lymphoid progenitors. The chronology of gene upregulation along the differentiation trajectories strongly suggests that specification and commitment to each lineage is not equivalent, but in stages of coordinated upregulation of genes. A list of top genes that were dynamically expressed across pseudotime ordering can be found in Table S2.

**ADDITIONAL DISCUSSION**

Our experiments are based on recent advances in molecular profiling technologies to provide at single-cell resolution on atlas of early blood stem cell differentiation in healthy human BM. Single cell approaches by definition better assess cell-cell heterogeneity than can in bulk population assays. As differentiation is asynchronous, scRNA-seq derived from a population of differentiating cells yields a snapshot representing the full dynamic ranges of these cell states. Additionally, as we simply isolated stem and progenitor cells, and CD38 by cell sorting based on the CD34 antigen in order to separate primitive from more mature cell types, we avoided constrained definitions of cells based on complex mixtures of cell surface antigens, themselves inferred from classic progenitor assays in semisolid medium. Also, in contrast to historic assays, we made efforts to limit physical manipulations, exposure to non-physiologic physical and chemical perturbation, and in vitro culture, all of which may alter a cell’s transcriptional program and other biologic and molecular characteristics. Thus, we can provide a resource for investigation of the earliest steps of human hematopoietic differentiation.

Early precursors residing proximate to the conceptual peak of hematopoietic hierarchy include long-term HSCs, short-term HSCs, and MPPs. However, the transcriptome of these phenotypically and functionally defined populations closely resemble each other. Further, despite profound differences in the self-renewal ability of HSCs and MLPs, the stem-cell program was partially carried over into MLPs [12]. A recent study that applied scRNA-seq to marrow HSPCs described the CD34^+^CD38^-^ compartment as a ‘continuum of low-primed undifferentiated hematopoietic stem and progenitor cells’ [8], stating the early HSPC continuum contains phenotypic MPPs not as discrete progenitor cell types but rather transitory states. In line with these results, we found the CD34^+^CD38^-^ progenitors were highly interconnected, and elements of stem, lymphoid, and myeloid programs were retained in MLPs, a group of primed CD34^+^CD38^-^ cells.

The hierarchical tree-like model of hematopoiesis describes a unique binary split between myeloid and lymphoid fates immediately downstream of multipotent cells, followed by a series of increasingly lineage-restricted oligopotent and unipotent progenitors. But those phenotypically defined populations like MPPs and CMPs were found to be heterogeneous and could be further purified; adult BM hematopoiesis is dominated by multipotent and unipotent progenitors, and megakaryocyte/erythroid activity are restricted to the stem cell compartment [29]. In our study, we derived a four-branch hematopoietic development trajectory with the earliest fate split separates erythroid-megakaryocyte progenitors from lymphoid-myeloid progenitors in human BM CD34^+^ cells without pre-purification of hematopoietic subpopulations, aligned with published scRNA-seq data [8, 30]. Lymphoid-myeloid progenitors further differentiated towards lymphocytes, granulocytes, dendritic cells and monocytes. Extended analysis of BM Lin^-^ cells by other researchers has revealed basophil branch emerges from CD135^-^ cells already committed toward a mixed MK/Erythroid/Basophil potential [30]. Taken together, these works suggest a refined hierarchy of human hematopoiesis, differ from the classical structure. It should be reminded that scRNA profiling generates a static snapshot of the transcriptional landscape and cannot provide conclusive information on the dynamics occurring along cell state transitions. Future efforts toward fate mapping in vitro and in vivo will be required for confirmation.

Recent studies using scRNA-seq and scATAC-seq profiling of sorted populations have suggested fate decisions in hematopoiesis are continuous [8, 10]. It is of importance to integrate different type of data in hematopoiesis to help understand the interaction between DNA and RNA. Though many studies used RNA to characterize normal hematopoiesis, Buenrostro’s work is the only one that utilize rigorous statistical approach to prove the coordination between mRNA and DNA-accessibility. We extended the analysis by including single-cell lncRNA and ATAC-seq data. The results not only confirmed Buenrostro’s findings, but also proved that lncRNA add a more coordinative layer to regulate hematopoiesis.

scRNA-seq provides new opportunities for discovery and characterization at the molecular levels of early HSC differentiation and developmental intermediates, retrospectively, and without the need to isolate purified populations. Deep analysis also provides a transcriptomic map of fate decisions taken by early progenitors. However, information inferred from scRNA-seq may be obscured due to missing reads and limited cell numbers. More cells would provide greater detail and higher resolution mapping. Given the low frequency of megakaryocyte progenitors within the CD34^+^ cells as well as the neglected Lin^-^CD34^-^ bone marrow compartment, we could not fully resolve the separation and maturation of all lineages. Nonetheless, we found good coverage of cell types and a similar HSPC Atlas as other published studies despite our limited number of starting cells. Our data accurately reflect the pattern of normal hematopoiesis, which may help to revise and refine characterization of hematopoiesis and provide a general reference framework to investigate the complexities of blood cell production at single-cell resolution – especially when cell numbers are limited, as from patient samples and in marrow failure syndromes.

1. Zhao, X., et al., *Single-cell RNA-seq reveals a distinct transcriptome signature of aneuploid hematopoietic cells.* Blood, 2017. **130**(25): p. 2762-2773.

2. Angerer, P., et al., *destiny: diffusion maps for large-scale single-cell data in R.* Bioinformatics, 2016. **32**(8): p. 1241-3.

3. Haghverdi, L., et al., *Diffusion pseudotime robustly reconstructs lineage branching.* Nat Methods, 2016. **13**(10): p. 845-8.

4. Choi, Y. and C. Kendziorski, *Statistical methods for gene set co-expression analysis.* Bioinformatics, 2009. **25**(21): p. 2780-6.

5. Scialdone, A., et al., *Computational assignment of cell-cycle stage from single-cell transcriptome data.* Methods, 2015. **85**: p. 54-61.

6. Graham, S.M., et al., *Transcriptional analysis of quiescent and proliferating CD34+ human hemopoietic cells from normal and chronic myeloid leukemia sources.* Stem Cells, 2007. **25**(12): p. 3111-20.

7. Whitfield, M.L., et al., *Identification of genes periodically expressed in the human cell cycle and their expression in tumors.* Mol Biol Cell, 2002. **13**(6): p. 1977-2000.

8. Velten, L., et al., *Human haematopoietic stem cell lineage commitment is a continuous process.* Nat Cell Biol, 2017. **19**(4): p. 271-281.

9. Zheng, S., et al., *Molecular transitions in early progenitors during human cord blood hematopoiesis.* Mol Syst Biol, 2018. **14**(3): p. e8041.

10. Buenrostro, J.D., et al., *Integrated Single-Cell Analysis Maps the Continuous Regulatory Landscape of Human Hematopoietic Differentiation.* Cell, 2018. **173**(6): p. 1535-1548 e16.

11. Ramskold, D., et al., *Full-length mRNA-Seq from single-cell levels of RNA and individual circulating tumor cells.* Nat Biotechnol, 2012. **30**(8): p. 777-82.

12. Laurenti, E., et al., *The transcriptional architecture of early human hematopoiesis identifies multilevel control of lymphoid commitment.* Nat Immunol, 2013. **14**(7): p. 756-63.

13. Chen, L., et al., *Transcriptional diversity during lineage commitment of human blood progenitors.* Science, 2014. **345**(6204): p. 1251033.

14. Pauklin, S. and L. Vallier, *The cell-cycle state of stem cells determines cell fate propensity.* Cell, 2013. **155**(1): p. 135-47.

15. Kastan, M.B. and J. Bartek, *Cell-cycle checkpoints and cancer.* Nature, 2004. **432**(7015): p. 316-23.

16. Nakamura-Ishizu, A., H. Takizawa, and T. Suda, *The analysis, roles and regulation of quiescence in hematopoietic stem cells.* Development, 2014. **141**(24): p. 4656-66.

17. Yang, J., et al., *Single cell transcriptomics reveals unanticipated features of early hematopoietic precursors.* Nucleic Acids Res, 2017. **45**(3): p. 1281-1296.

18. Suda, T., K. Takubo, and G.L. Semenza, *Metabolic regulation of hematopoietic stem cells in the hypoxic niche.* Cell Stem Cell, 2011. **9**(4): p. 298-310.

19. Yu, W.M., et al., *Metabolic regulation by the mitochondrial phosphatase PTPMT1 is required for hematopoietic stem cell differentiation.* Cell Stem Cell, 2013. **12**(1): p. 62-74.

20. Wolf, F.A., et al., *PAGA: graph abstraction reconciles clustering with trajectory inference through a topology preserving map of single cells.* Genome Biol, 2019. **20**(1): p. 59.

21. Orkin, S.H. and L.I. Zon, *Hematopoiesis: an evolving paradigm for stem cell biology.* Cell, 2008. **132**(4): p. 631-44.

22. Adolfsson, J., et al., *Identification of Flt3+ lympho-myeloid stem cells lacking erythro-megakaryocytic potential a revised road map for adult blood lineage commitment.* Cell, 2005. **121**(2): p. 295-306.

23. Galy, A., et al., *Human T, B, natural killer, and dendritic cells arise from a common bone marrow progenitor cell subset.* Immunity, 1995. **3**(4): p. 459-73.

24. Mikkola, H.K., et al., *Haematopoietic stem cells retain long-term repopulating activity and multipotency in the absence of stem-cell leukaemia SCL/tal-1 gene.* Nature, 2003. **421**(6922): p. 547-51.

25. Orkin, S.H., et al., *Transcription factor GATA-1 in megakaryocyte development.* Stem Cells, 1998. **16 Suppl 2**: p. 79-83.

26. Shivdasani, R.A. and S.H. Orkin, *The transcriptional control of hematopoiesis.* Blood, 1996. **87**(10): p. 4025-39.

27. Akashi, K., et al., *A clonogenic common myeloid progenitor that gives rise to all myeloid lineages.* Nature, 2000. **404**(6774): p. 193-7.

28. Terskikh, A.V., et al., *Gene expression analysis of purified hematopoietic stem cells and committed progenitors.* Blood, 2003. **102**(1): p. 94-101.

29. Notta, F., et al., *Distinct routes of lineage development reshape the human blood hierarchy across ontogeny.* Science, 2016. **351**(6269): p. aab2116.

30. Pellin, D., et al., *A comprehensive single cell transcriptional landscape of human hematopoietic progenitors.* Nat Commun, 2019. **10**(1): p. 2395.
