# Supplementary material for: Comprehensive analysis of single-cell RNA sequencing data from healthy human marrow hematopoietic cells
Source: BMC Res Notes. 2020 Nov 10;13:514. doi: 10.1186/s13104-020-05357-y (PMC7653854; doi:10.1186/s13104-020-05357-y)
Supplement: Supplementary file 2 — Additional file 2: Figure S1. (A) t-distributed Stochastic Neighbor Embedding (tSNE) plot of single-cell gene expression data. Cells were labeled according to assigned cell types. (B) Assignment of a HSPC type to each cluster based on the significance of overlapping between HSPC- and cluster-specific genes (Fisher’s exact test). (C) Proportions of HSCs, MLPs, GMPs, ProBs, ETPs, and MEPs in each of the cell cycle categories. Cell types displayed were based on the tSNE results. Figure S2. (A, B) Visualization of the HSPC continuum. Cells were colored based on FACS sorting surface marker CD38 in (A). Clusters 2 and 6, and undefined cells were hidden to show Cluster 3 clearly in (B). (C) Expression levels of immunophenotypic populations based on surface markers were overlaid on the cellular hierarchy. (D) Enriched GO terms of differentially expressed genes in ETPs. Figure S3. Visualization of the HSPC continuum. Ordering of individual cells into a three-dimensional independent component space of hematopoietic lineages using a diffusion map. Each ball represents one cell. Cells were colored based on different clusters defined from Fig. 1c in Panel A. (B) PCA of single-cell gene expression data. Cells were labeled according to assigned cell types. (C) Partition-based graph abstraction generated a topology-preserving map of single cells. Nodes correspond to cell groups and edge weights quantifies the connectivity between groups. Figure S4. Large-scale shifts in gene expression during development of hematopoietic cells. (A) Global analysis of gene expression kinetics along the trajectory identified genes that varied significantly over pseudotime development. Bars on top indicate locations of individual cells, colored by stages of development, along this developmental trajectory. (B) Enriched GO terms of differentially expressed genes in each population. Figure S5. Reconstructing the topology of early fate decisions. (A) Expression levels of hematopoietic transcriptional fac [file 13104_2020_5357_MOESM2_ESM.pptx]

## Slide 1
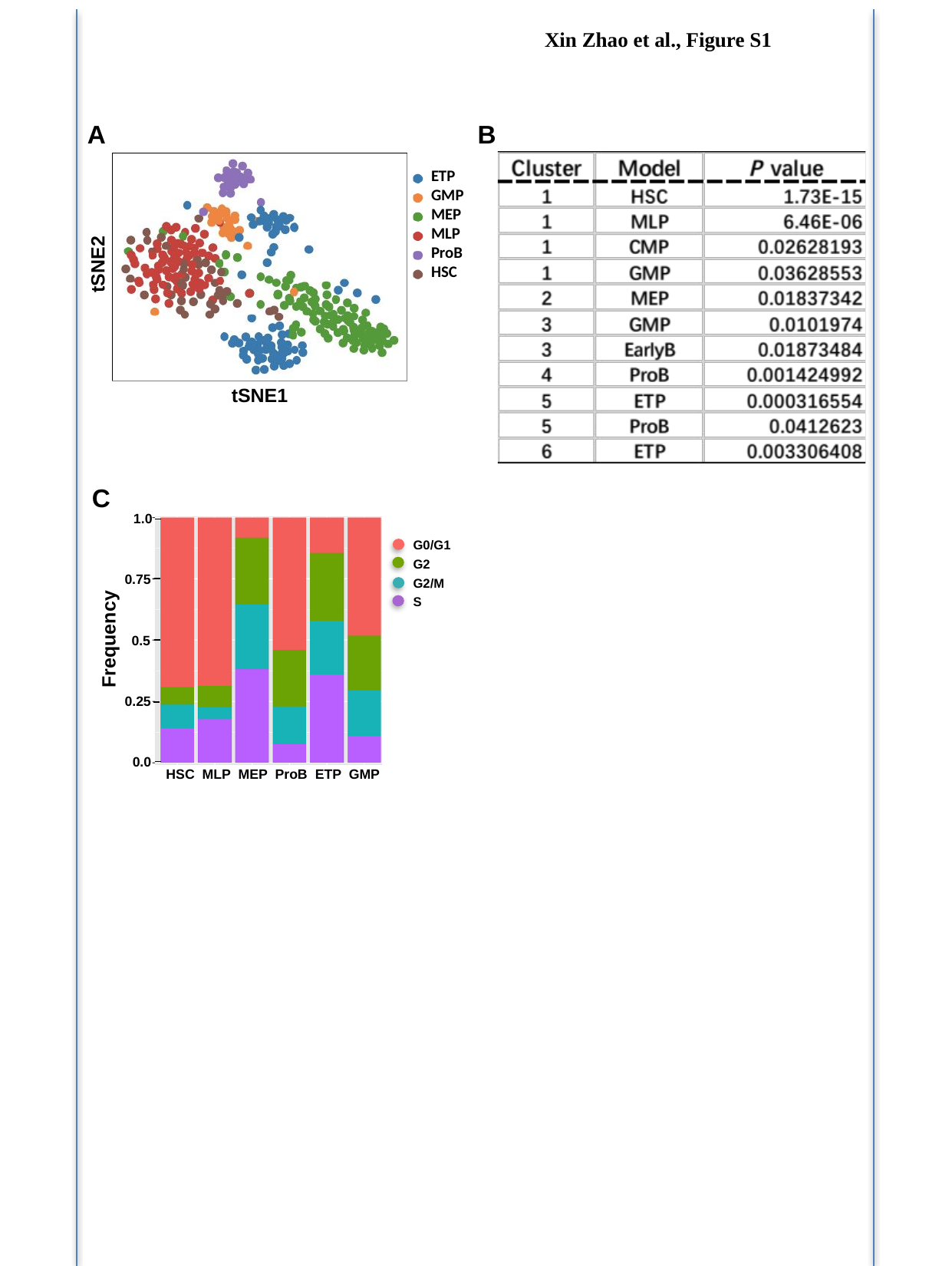

Xin Zhao et al., Figure S1
A
ETP
GMP
MEP
MLP
ProB
HSC
tSNE2
tSNE1
B
C
1.0
G0/G1
G2
G2/M
S
0.75
Frequency
0.5
0.25
0.0
HSC MLP MEP ProB ETP GMP

## Slide 2
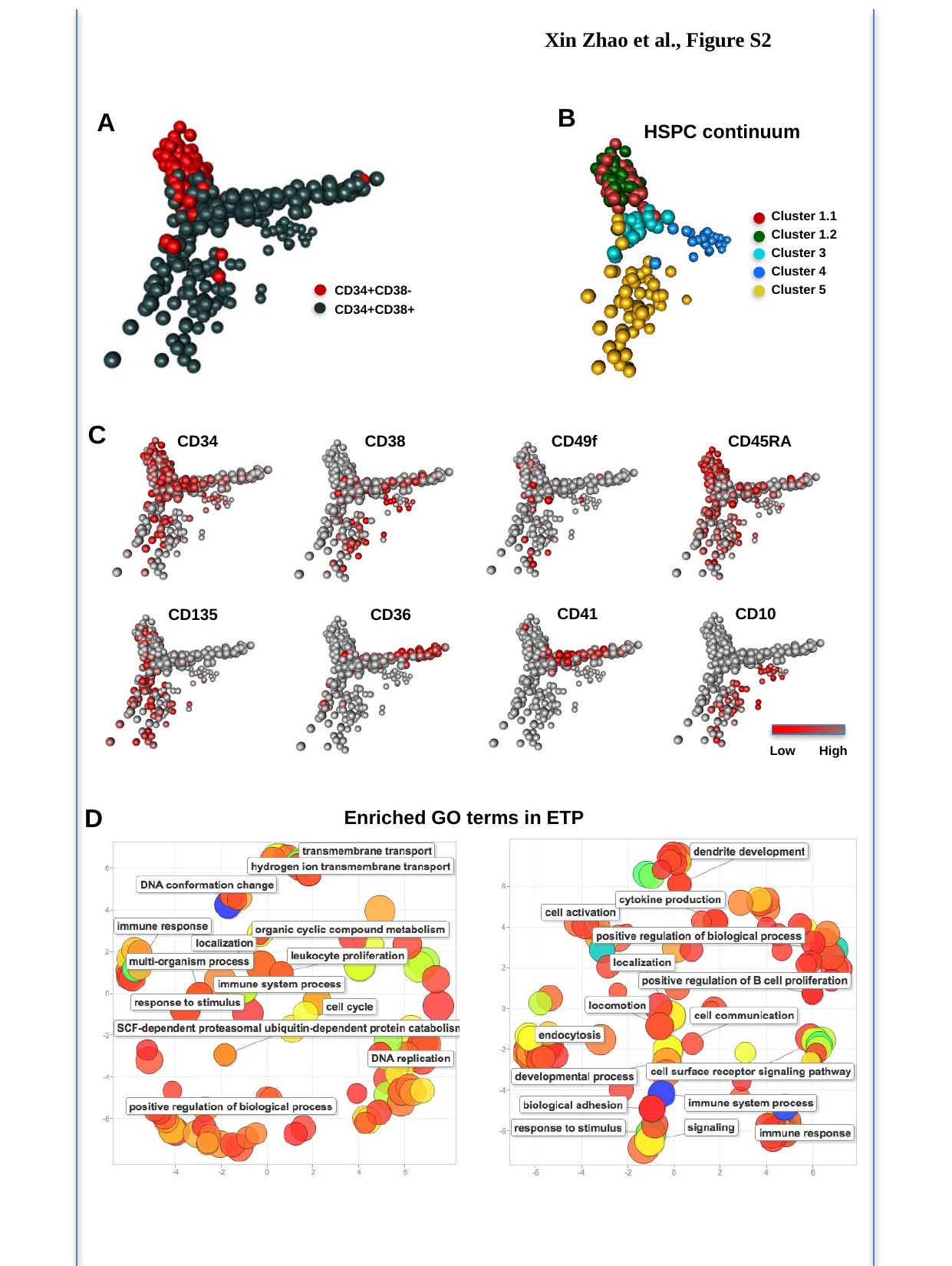

Xin Zhao et al., Figure S2
B
HSPC continuum
Cluster 1.1
Cluster 1.2
Cluster 3
Cluster 4
Cluster 5
A
CD34+CD38-
CD34+CD38+
C
CD34
CD38
CD49f
CD45RA
CD135
CD41
CD10
Low
High
CD36
D
Enriched GO terms in ETP

## Slide 3
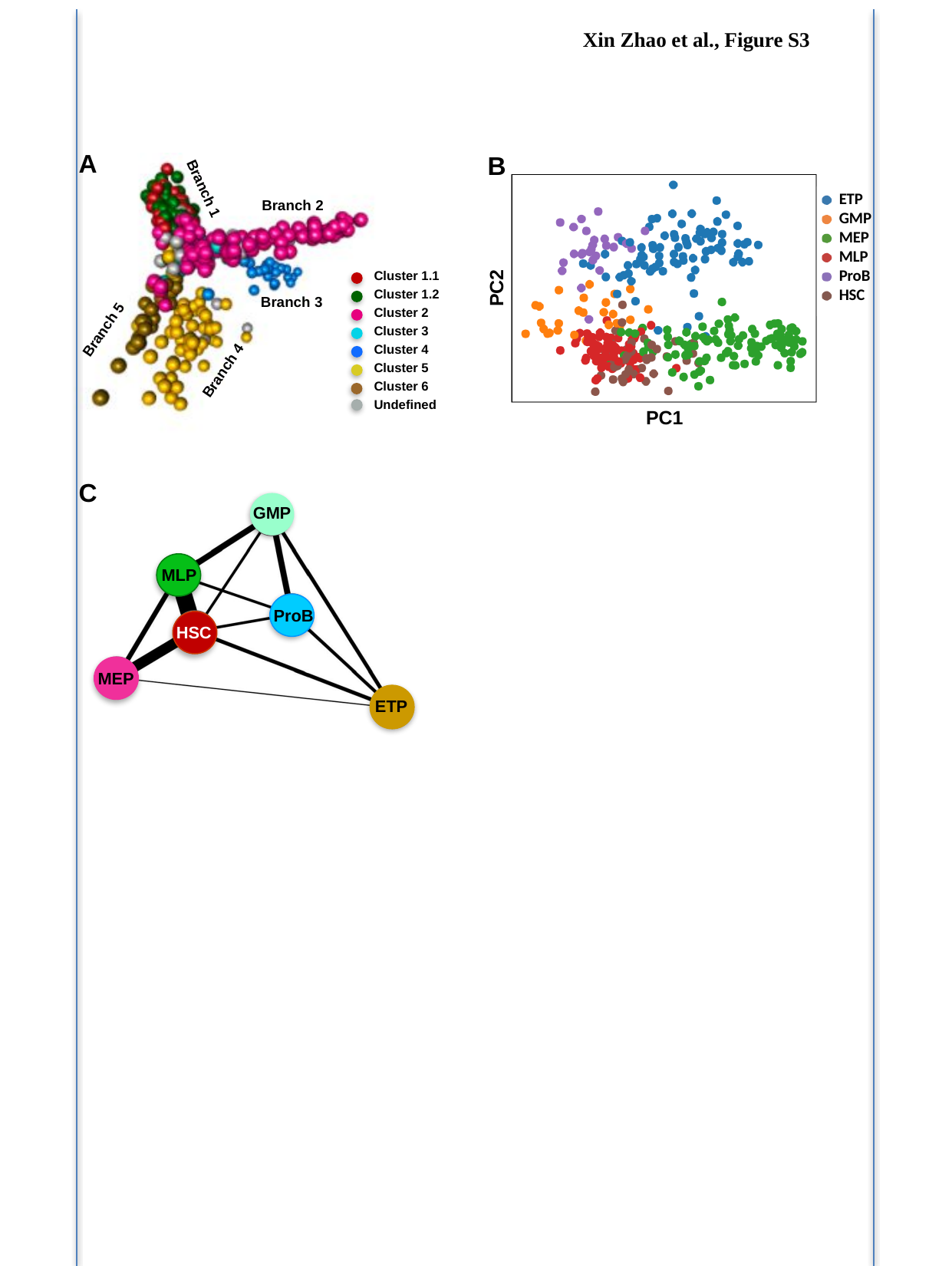

Xin Zhao et al., Figure S3
A
Branch 1
Branch 2
Cluster 1.1
Cluster 1.2
Cluster 2
Cluster 3
Cluster 4
Cluster 5
Cluster 6
Undefined
Branch 3
Branch 5
Branch 4
B
PC2
PC1
ETP
GMP
MEP
MLP
ProB
HSC
C
GMP
MLP
ProB
HSC
MEP
ETP

## Slide 4
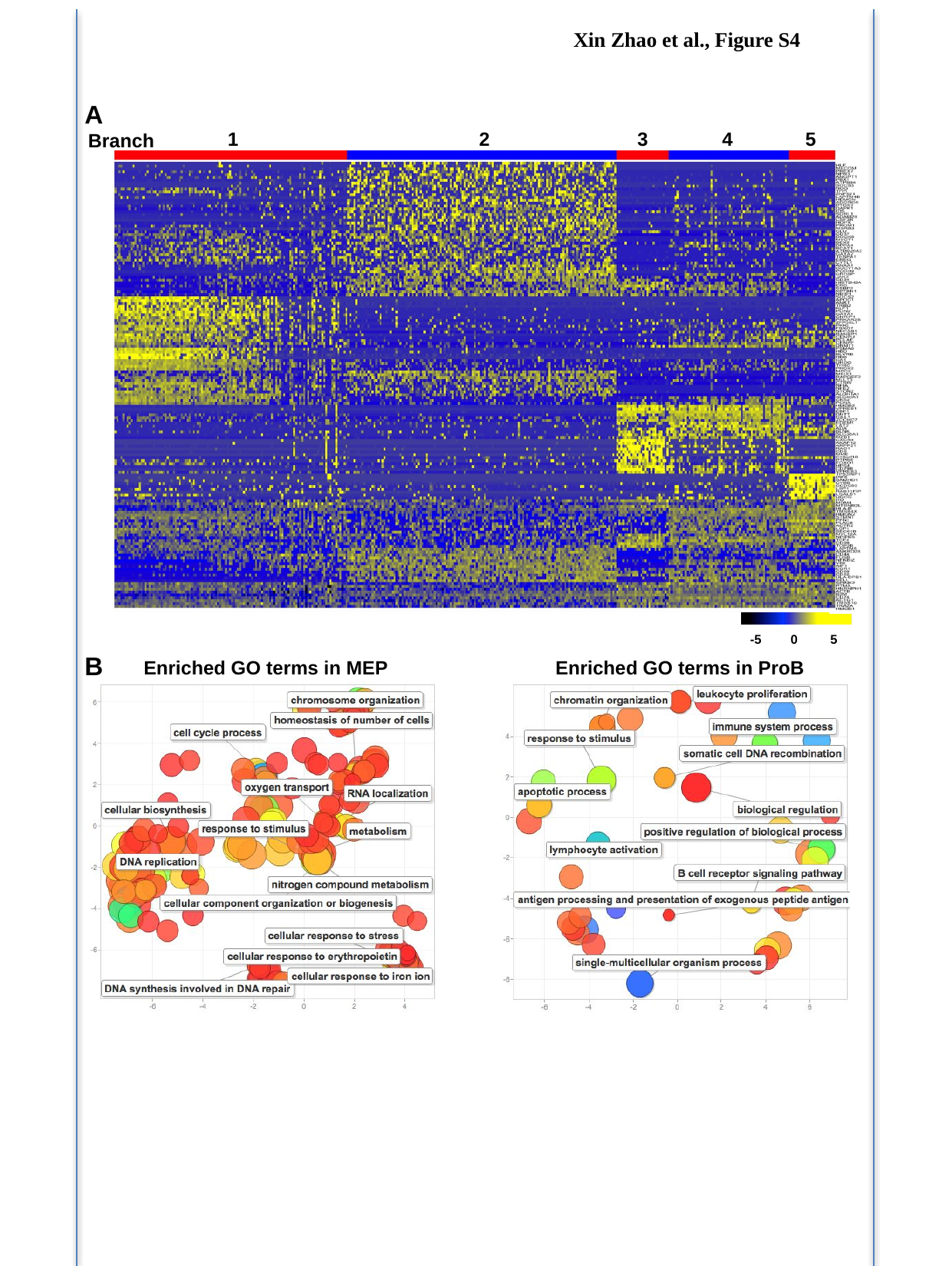

Xin Zhao et al., Figure S4
A
2
3
4
5
1
Branch
-5 0 5
B
Enriched GO terms in MEP
Enriched GO terms in ProB

## Slide 5
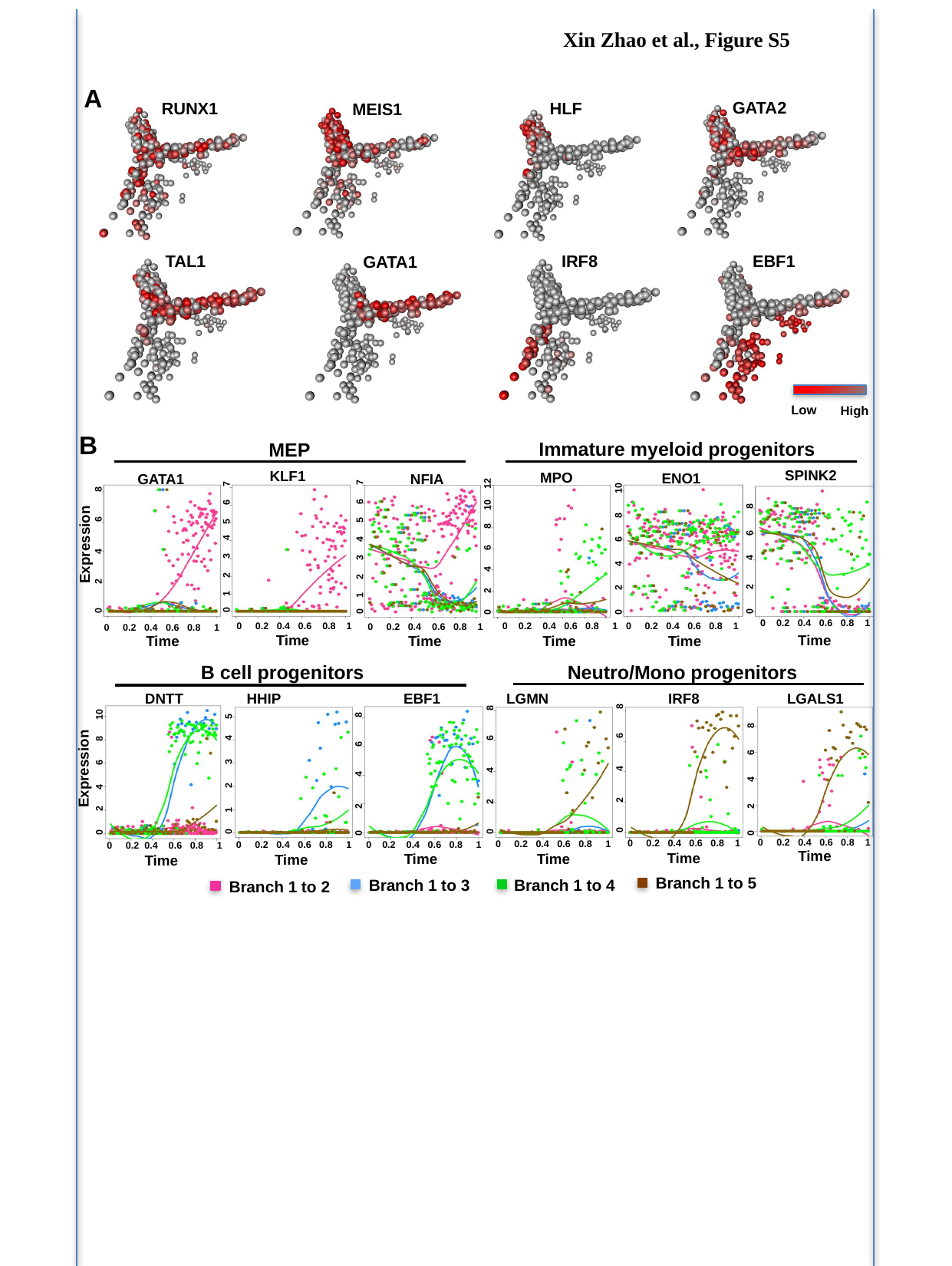

Xin Zhao et al., Figure S5
A
GATA2
HLF
RUNX1
MEIS1
IRF8
EBF1
TAL1
GATA1
Low
High
B
Immature myeloid progenitors
MEP
SPINK2
0 2 4 6 8
0 0.2 0.4 0.6 0.8 1
KLF1
0 1 2 3 4 5 6 7
0 0.2 0.4 0.6 0.8 1
MPO
ENO1
0 2 4 6 8 10
0 0.2 0.4 0.6 0.8 1
GATA1
NFIA
0 0.2 0.4 0.6 0.8 1
0 2 4 6 8
Expression
0 2 4 6 8 10 12
0 1 2 3 4 5 6 7
0 0.2 0.4 0.6 0.8 1
0 0.2 0.4 0.6 0.8 1
Time
Time
Time
Time
Time
Time
B cell progenitors
Neutro/Mono progenitors
LGMN
LGALS1
IRF8
DNTT
HHIP
EBF1
Expression
0 2 4 6 8 10
0 2 4 6 8
0 2 4 6 8
0 2 4 6 8
0 1 2 3 4 5
0 2 4 6 8
0 0.2 0.4 0.6 0.8 1
0 0.2 0.4 0.6 0.8 1
0 0.2 0.4 0.6 0.8 1
0 0.2 0.4 0.6 0.8 1
0 0.2 0.4 0.6 0.8 1
0 0.2 0.4 0.6 0.8 1
Time
Time
Time
Time
Time
Time
Branch 1 to 5
Branch 1 to 4
Branch 1 to 3
Branch 1 to 2

## Slide 6
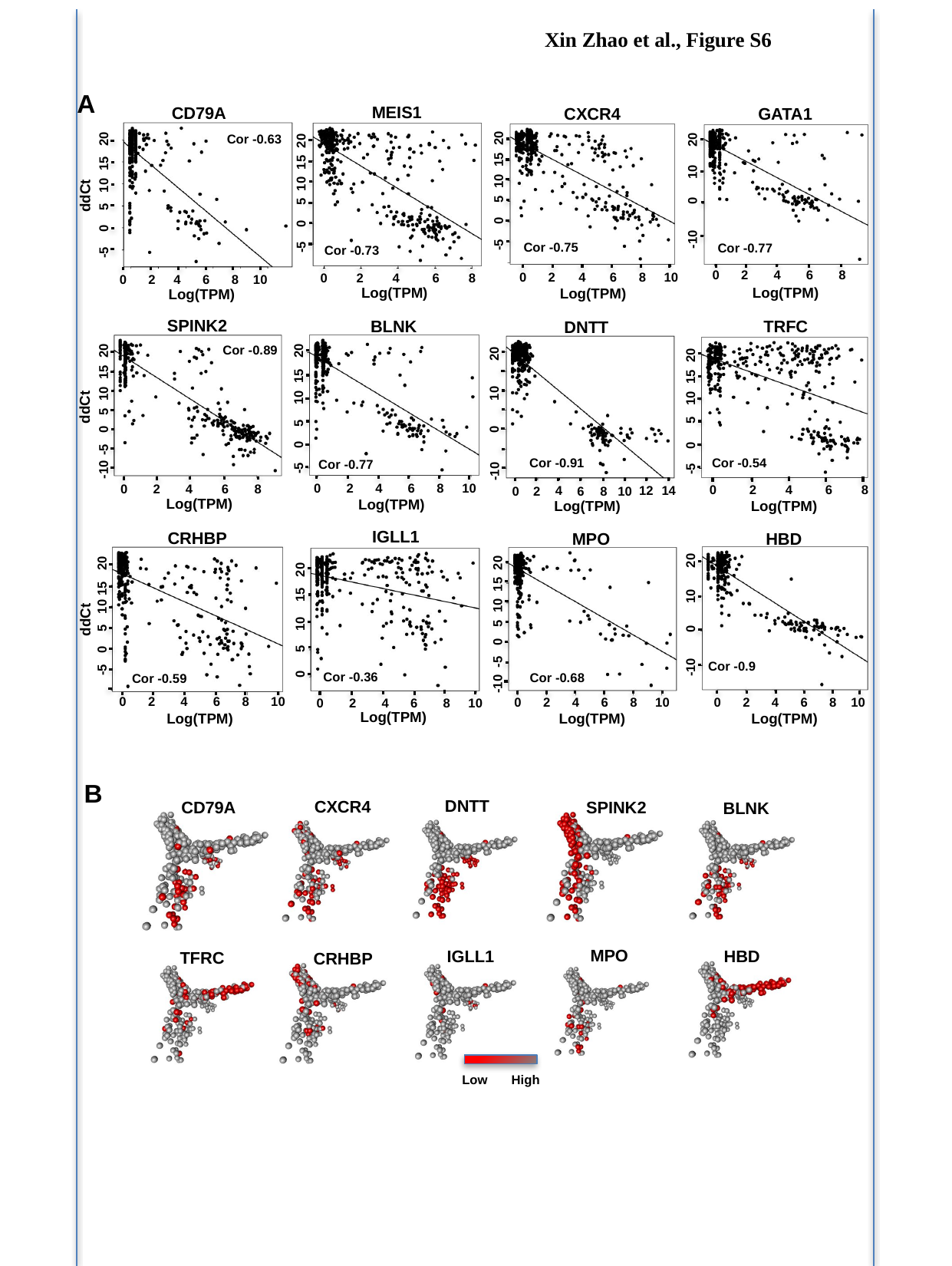

Xin Zhao et al., Figure S6
MEIS1
-5 0 5 10 15 20
Cor -0.73
0 2 4 6 8
Log(TPM)
CXCR4
-5 0 5 10 15 20
Cor -0.75
Log(TPM)
A
CD79A
Cor -0.63
-5 0 5 10 15 20
ddCt
Log(TPM)
-10 0 10 20
 Cor -0.77
GATA1
0 2 4 6 8
Log(TPM)
 0 2 6 8 10
4
0 2 4 6 8 10
BLNK
-5 0 5 10 15 20
Cor -0.77
0 2 4 6 8 10
Log(TPM)
TRFC
-5 0 5 10 15 20
 Cor -0.54
0 2 4 6 8
Log(TPM)
14
 -5 0 5 10 15 20
-10
Cor -0.89
ddCt
Log(TPM)
SPINK2
DNTT
 0 10 20
Cor -0.91
-10
0 2 4 6 8
12
4
 8
 10
 6
0
2
Log(TPM)
IGLL1
0 5 10 15 20
Cor -0.36
0 2 4 6 8 10
Log(TPM)
HBD
-10 0 10 20
 Cor -0.9
0 2 4 6 8 10
Log(TPM)
CRHBP
-5 0 5 10 15 20
ddCt
Cor -0.59
0 2 4 6 8 10
Log(TPM)
MPO
-10 -5 0 5 10 15 20
Cor -0.68
 0 2 6 8 10
4
Log(TPM)
B
DNTT
CXCR4
SPINK2
CD79A
BLNK
MPO
HBD
IGLL1
TFRC
CRHBP
Low
High

## Slide 7
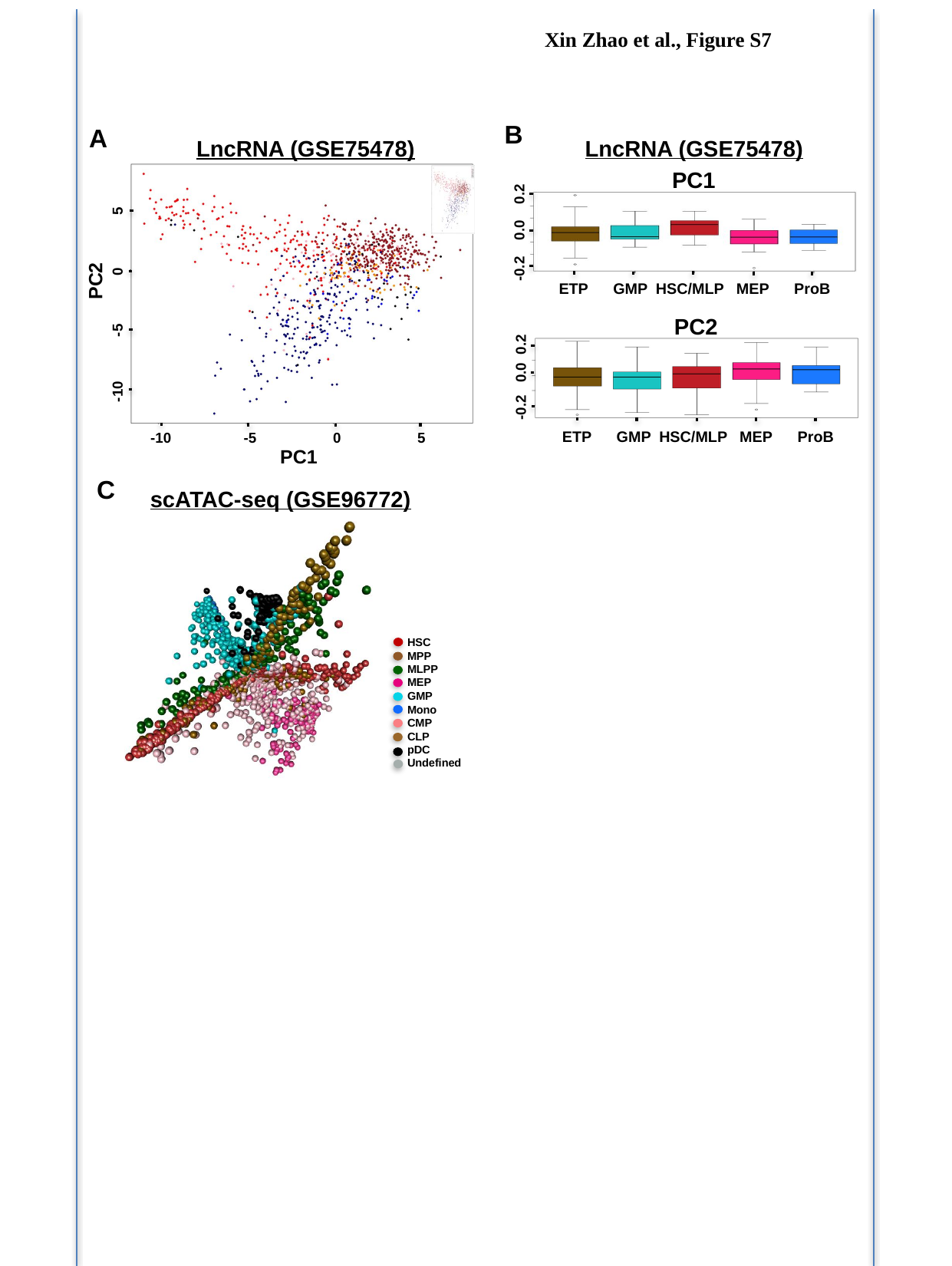

Xin Zhao et al., Figure S7
B
PC1
-0.2 0.0 0.2
ETP GMP HSC/MLP MEP ProB
LncRNA (GSE75478)
A
PC2
-10 -5 0 5
-10 -5 0 5
PC1
LncRNA (GSE75478)
PC2
-0.2 0.0 0.2
 ETP GMP HSC/MLP MEP ProB
C
scATAC-seq (GSE96772)
HSC
MPP
MLPP
MEP
GMP
Mono
CMP
CLP
pDC
Undefined
